# Sweet’s Syndrome: A First in Human Lung Transplantation

**DOI:** 10.1016/j.chest.2021.04.018

**Published:** 2021-08-05

**Authors:** Allison L. Ramsey, W. Dean Wallace, Fereidoun Abtin, Jeffrey D. Suh, Lloyd L. Liang, Sapna Shah, Joseph P. Lynch, John Belperio, Ariss Derhovanessian, Ian Britton, David M. Sayah, Michael Y. Shino, S. Sam Weigt, Rajan Saggar

**Affiliations:** a Division of Pulmonary and Critical Care Medicine, University of California, Los Angeles David Geffen School of Medicine, Los Angeles, CA; b Department of Pathology, Keck School of Medicine of the University of Southern California, Los Angeles, CA; c Thoracic Imaging at the Department of Radiological Sciences, David Geffen School of Medicine, UCLA Health System, Los Angeles, CA; d Department of Otolaryngology-Head and Neck Surgery, University of California Los Angeles, Los Angeles, CA

**Keywords:** acute febrile neutrophilic dermatosis, lung transplantation, Sweet’s syndrome, HRCT, high-resolution CT, LT, lung transplantation, PFT, pulmonary function test, SS, Sweet’s syndrome

## Abstract

Sweet’s Syndrome (SS), also known as acute febrile neutrophilic dermatosis, is one of several cutaneous inflammatory disorders classified as neutrophilic dermatoses. Respiratory complications are described in <50 cases in the literature,^1^ without prior report of lung transplantation (LT). This article explains the clinical course of the first patient to receive LT for pulmonary SS and presents evidence suggesting recurrence of the primary lung disease in the allograft.

## Case Report

A 36-year-old woman was evaluated in our LT clinic for hypoxemia and respiratory failure due to obstructive lung disease. Seven years prior, she developed fever and tender skin papules demonstrating dense neutrophilic dermal infiltration, without vasculitis or immunobullous disease on skin biopsy. The clinical presentation and swift response to corticosteroid therapy fulfilled diagnostic criteria for SS.^2^ She developed cough, dyspnea, and rhinosinusitis 4 years before, at which time results of pulmonary function tests (PFTs) were normal (FVC, 4.16 L [98%]; FEV_1_, 3.37 L [95%]). She required extensive sinus surgery 4 years 5 months before presentation. High-resolution CT (HRCT) of the chest demonstrated diffuse airway thickening, mosaic attenuation, and bronchocentric nodularity, consistent with small/large airways disease. Despite multiple unremarkable infectious evaluations of lower respiratory tract samples, she was treated with repeated courses of antibiotics/corticosteroids.

**Figure 1:**
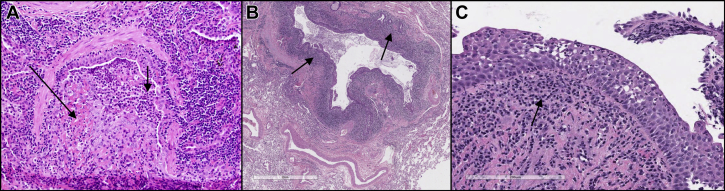
A, Bronchiole with severe mucosal and luminal neutrophilic inflammation (short arrow) and early organizing pneumonia (long arrow), including fibrin deposition, based on surgical lung biopsy performed 19 months before lung transplantation for Sweet’s Syndrome (hematoxylin and eosin stain; original magnification 200×). B, Explant lung pathology showing diffuse bronchiectasis with exuberant airway inflammation (arrow), including frequent neutrophils in a patient with Sweet’s syndrome (hematoxylin and eosin stain; original magnification 20×). C, Cryobiopsy of lung allograft (22 months after transplantation) during an acute flare of Sweet’s syndrome showing dense mixed inflammation (arrow), including numerous neutrophils involving the bronchial mucosa (hematoxylin and eosin stain; original magnification 200×).

After the onset of abnormal pulmonary function and new oxygen requirements, she underwent surgical lung biopsy 15 months before presentation, revealing marked airway-centric organizing pneumonia with dense neutrophilic infiltration (Fig 1A). Two months after presentation, PFT showed severe obstruction, air trapping, and loss of diffusing capacity (FVC, 1.94 L [42%]; FEV_1_, 0.92 L [24%]; total lung capacity 5.56 L [86%]; residual volume, 3.62 L [165%]; diffusing capacity for carbon monoxide, 10.7 mL/min/mm Hg [35%]). She underwent bilateral LT 4 months after presentation, and 4.5 years after the onset of respiratory symptoms. Explant pathology showed severe bronchiectasis/bronchiolectasis with mixed chronic airway inflammation, including numerous neutrophils, but devoid of residual organizing pneumonia (Fig 1B). The post-LT immunosuppression/immunomodulation included rabbit anti-thymocyte globulin, tacrolimus, mycophenolate mofetil, prednisone, and azithromycin.

The post-LT course was complicated by multiple sinus surgeries for possible fungal sinusitis; however, there was never pathologic evidence for sinus invasion/necrosis/bone involvement. Beginning 8 months post-LT, the patient experienced recurrent hospitalizations for fever, productive cough, sinus congestion/pain, and dyspnea. At 10 months post-LT, the patient manifested worsening respiratory symptomatology, a new obstructive ventilatory defect on PFT, and HRCT chest findings consistent with mild bilateral diffuse small/large airway thickening, and multifocal centrilobular nodules, without air trapping. Bronchoscopy demonstrated a neutrophil-predominant (70%) BAL cell count, and a sterile neutrophilic bronchopneumonia on transbronchial biopsy, without evidence for acute rejection. The patient was treated with methylprednisolone (3 mg/kg) with immediate clinical improvement. Subsequently, a slow prednisone (1 mg/kg) wean resulted in resolution of the obstructive ventilatory defect at 13 months post-LT. Extrapulmonary activity also abated and included ophthalmologic (bilateral blepharitis and central serous chorioretinopathy) and musculoskeletal (myositis on muscle biopsy) manifestations.

Despite the slow weaning of corticosteroids, her respiratory symptom complex relapsed, and SS skin lesions reappeared on the bilateral forearms. The chest HRCT showed progression of diffuse small/large airway thickening (including tracheal/mainstem involvement) and air trapping. Eventually, patchy and nodular/confluent centrilobular ground-glass infiltrates evolved, with new oxygen requirements consistent with superimposed multifocal pneumonia, despite repeatedly negative BAL microbiology. These findings prompted a cryobiopsy and tracheal biopsy (22 months post-LT), which highlighted severe neutrophilic inflammation of the bronchial/tracheal mucosa (Fig 1C), with a neutrophil-predominant (90%) BAL cell count and negative microbiology.

**Figure 2:**
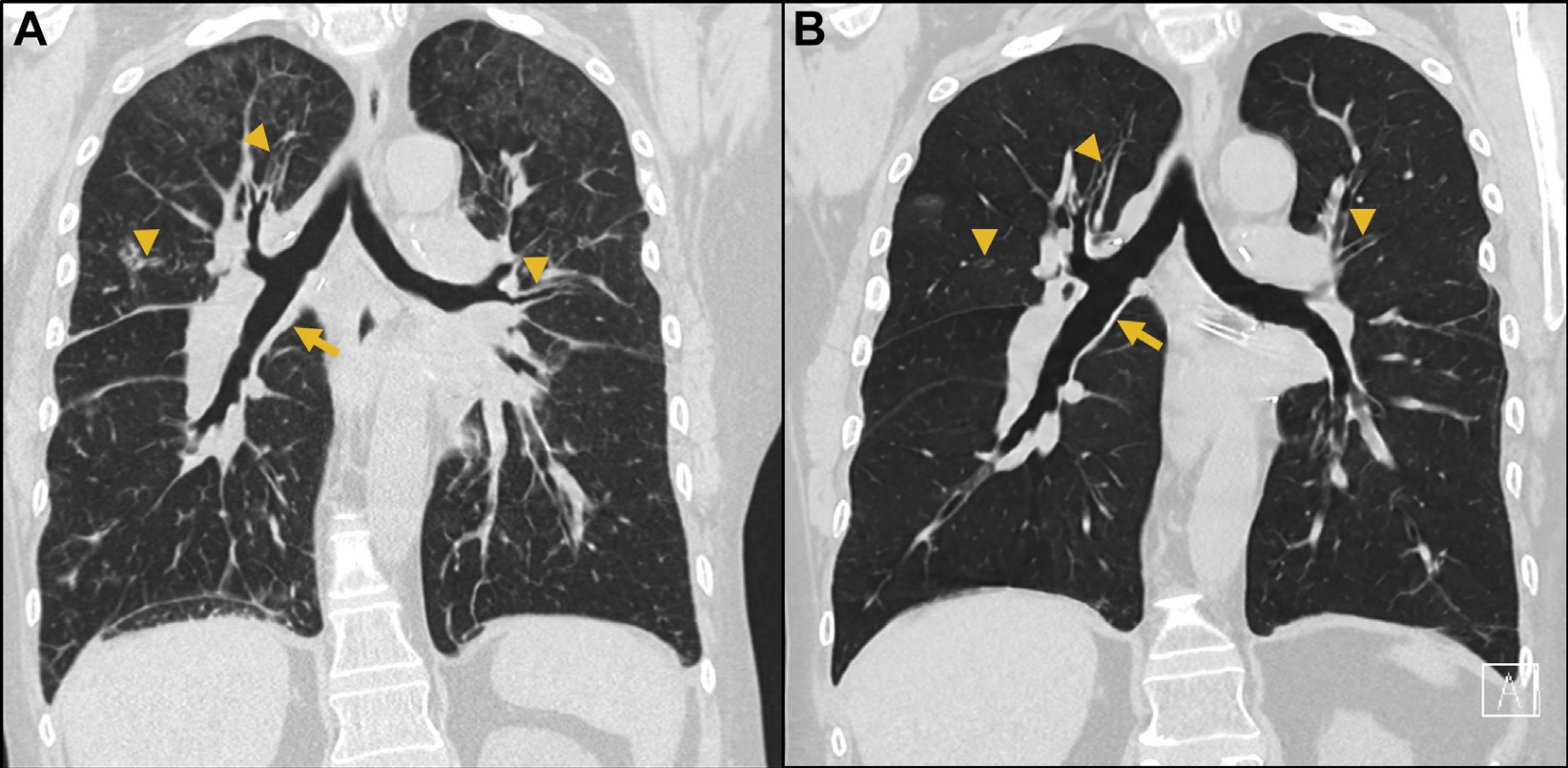
A, HRCT chest coronal reconstruction (22 months after lung transplantation for Sweet’s syndrome), highlighting diffuse small and large airway wall thickening involving the bronchus intermedius (arrow) and bilateral segmental airways (arrowheads). Multifocal and confluent ground-glass infiltrates are best visualized in the bilateral upper lobes. B, HRCT chest coronal reconstruction (23 months after lung transplantation for Sweet’s syndrome), highlighting the relatively rapid resolution of small and large airway wall thickening involving the bronchus intermedius (arrow) and bilateral segmental airways (arrowheads) after augmented corticosteroid therapy. In addition, the upper lobe predominant alveolar infiltrates demonstrate near resolution. HRCT = high-resolution CT.

Systemic corticosteroids were administered for presumed recurrence of pulmonary SS with prompt improvement of respiratory symptoms, HRCT chest infiltrates (Figs 2A, 2B), and skin lesions. Rilonacept (IL-1 receptor antagonist) was introduced to promote disease quiescence. At 4 years post-LT, pulmonary function is preserved (FVC, 3.28 [71%]; FEV_1_, 2.60 [70%]) without evidence for chronic lung allograft dysfunction.

## Discussion

The patient met diagnostic criteria for classical SS set forth by von den Driesch in 1994,^2^ satisfying both major and two of four minor criteria. Pulmonary involvement is rare and, unlike in this case, is generally associated with underlying hematologic disorders and radiographic/clinical resolution.^3^ Thoracic imaging findings generally range from alveolar/interstitial/nodular infiltrates in a unilateral/bilateral distribution, with or without pleural effusion.^3^ Pathologic findings include a neutrophil-predominant (>50%) BAL cell count, neutrophilic-infiltrating interstitial/airspace pneumonia, or organizing pneumonia. Although this patient’s pre-LT pathology of organizing pneumonia is consistent with reported cases,^4^ the explant pathology showed extensive bronchiectasis, likely representing the consequences of long-standing inflammatory airway disease.

The proposed post-LT recurrence of pulmonary SS is a diagnosis of exclusion. This notion is supported by sterile HRCT chest infiltrates, prominent neutrophilic infiltration of allograft biopsies, neutrophil-predominant BAL cell count, recurrent skin lesions, and predictable corticosteroid responsiveness. The comparison of the pre-LT with post-LT temporal progression of pulmonary SS gauged by sequential HRCT chest imaging (diffuse airway disease followed by airspace infiltration) and PFT assessments (progressive obstructive lung disease) is strikingly similar, further supporting recurrent pulmonary SS. SS joins a short list of recurrent primary lung diseases after LT, which includes sarcoidosis,^5^ lymphangioleiomyomatosis,^6^ hard-metal disease,^7^ and pulmonary capillary hemangiomatosis.^8^

Neutrophilic dermatoses have been suggested to have multiple overlapping clinical, genetic, and pathologic characteristics with established autoinflammatory diseases.^9^ Central to this concept are the consequences of uninhibited mediators of innate immunity (IL-1β, tumor necrosis factor-alpha, IL-17) and the potential for systemic disease resulting from tissue infiltration of dysregulated mature neutrophils. In addition, several preliminary lines of evidence support the role of the inflammasome and downstream IL-1 biology in solid organ and stem cell transplantation.^10^ As such, rilonacept may have contributed to the improvement and maintenance of pulmonary SS remission, as well as the avoidance of alloimmune injury after LT.^11^

This report highlights the first human LT for SS. We posit that pulmonary SS is primarily an airway disorder with obstructive lung disease, which may progress to end-stage bronchiectasis and require LT. We provide evidence for the probable recurrence of pulmonary SS in the allograft. Nevertheless, LT should be a careful consideration for pulmonary SS, perhaps in concert with IL-1 receptor blockade, which may attenuate the recurrence or progression of pulmonary SS in the post-LT setting.
